# Planning an artificial intelligence diabetic retinopathy screening program: a human-centered design approach

**DOI:** 10.3389/fmed.2023.1198228

**Published:** 2023-07-07

**Authors:** Angelica C. Scanzera, Cameron Beversluis, Archit V. Potharazu, Patricia Bai, Ariel Leifer, Emily Cole, David Yuzhou Du, Hugh Musick, R. V. Paul Chan

**Affiliations:** ^1^Department of Ophthalmology and Visual Sciences, Illinois Eye and Ear Infirmary, University of Illinois Chicago, Chicago, IL, United States; ^2^Institute for Healthcare Delivery Design, Office of Population Health Sciences, University of Illinois Chicago, Chicago, IL, United States; ^3^Department of Family and Community Medicine, University of Illinois at Chicago, Chicago, IL, United States; ^4^W.K. Kellogg Eye Center, University of Michigan, Ann Arbor, MI, United States; ^5^Segal Design Institute, Northwestern University, Evanston, IL, United States

**Keywords:** diabetic retinopathy, screening, artificial intelligence, human-centered design, implementation

## Abstract

Diabetic retinopathy (DR) is a leading cause of vision loss in the United States and throughout the world. With early detection and treatment, sight-threatening sequelae from DR can be prevented. Although artificial intelligence (AI) based DR screening programs have been proven to be effective in identifying patients at high risk of vision loss, adoption of AI in clinical practice has been slow. We adapted the United Kingdom Design Council’s Double-Diamond model to design a strategy for care delivery which integrates an AI-based screening program for DR into a primary care setting. Methods from human-centered design were used to develop a strategy for implementation informed by context-specific barriers and facilitators. The purpose of this community case study is to present findings from this work in progress, including a system of protocols, educational documents and workflows created using key stakeholder input.

## Introduction

Diabetic retinopathy (DR) is the leading cause of blindness globally in working-aged adults (1). In adults with diabetes, the global prevalence of DR and vision-threatening DR (which include severe non-proliferative DR, proliferative DR, and diabetic macular edema) is estimated to be 35% and 10%, respectively (2). Direct medical costs for DR in the U.S. were estimated to be $493 million in 2004, with rates on the rise (3). While early detection and treatment can prevent visual impairment by as much as 90% (4), fewer than half of Americans with diabetes receive a recommended yearly screening for eye disease (5–8).

### Telemedicine and artificial intelligence

Telemedicine has been proven to be a cost-effective method to screen patients for DR (3, 9, 10). With this option, patients with diabetes have retinal photos taken within a convenient location, often a primary care clinic, and images are communicated to an image reader for grading. Patients with pathology on image screening are then referred for timely in-person eye care. The National Health Service in the United Kingdom achieved screening rates over 80% using this technology, with diabetes no longer the leading cause of blindness in the U.K. for the first time in over 50 years (11–13). While telemedicine has gained increased acceptance since the onset of the COVID-19 pandemic (14, 15), the United States has been slower to adopt it despite efforts to use this technology to screen for DR (16–18).

Artificial intelligence (AI) offers a unique opportunity to assist in several areas within healthcare, including triage, disease screening, and management. Eric Topol, a cardiologist and researcher known for authoring *Deep Medicine: How Artificial Intelligence Can Make Healthcare Human Again* (2019), previously shared that ophthalmology, not radiology, is leading the AI movement (19). AI algorithms have been described for image analysis in retinal diseases including DR, age-related macular degeneration, retinopathy of prematurity, retinal vascular occlusions, and retinal detachment; AI has also been described for use in glaucoma, keratoconus, cataract, refractive error, intraocular lens power calculations, and when planning strabismus surgeries (20–22).

In DR screening, there is a promising role for the use of AI as a tool to identify patients at risk of vision loss. The IDx-DR AI and EyeArt AI (EyeNuk, Inc., Woodland Hills, CA) systems for DR screening have both received U.S. Food and Drug Administration authorization (23, 24). Using these technologies, photos are taken similarly to telemedicine programs and DR is detected by AI with a high level of accuracy. Liu et al. found that implementation of an automated DR screening program in a primary care clinic within a low-income metropolitan population improved adherence to follow-up eye care from 18 to 55% in those with referable findings (25). While studies show promising results, uptake of these systems continue to be slow.

Gunasekeran and Tham et al. illustrated the operationalization of new models of digital care in ophthalmology since the onset of the pandemic, which accelerated the adoption of new models of care (26). This community case study is a good example of the operational “pyramid” model. The pyramid model helps to sort or triage patients into three pathways: self-management or observation, remote video-consultation, or referral for in-person secondary or tertiary care (26–28). While the pyramid model provides benefits in triaging patients for referrals according to DR severity, it also requires significant clinical capacity and willingness to adopt AI into clinical practice (26). What has been learned from examples describing implementation of digital health in ophthalmology is that making new treatments or technologies available does not guarantee their adoption. In fact, it is estimated that original research can take up to 17 years to turn into patient care benefits (29). Implementation is never as simple as purchasing software or equipment. Systems need to be in place for the screening to be included within operational workflows and stakeholders must be involved and motivated to implement this service.

### Health disparities

Racial and ethnic minorities have a greater prevalence of vision threatening DR (2), and are at an increased risk of developing more severe DR than non-Hispanic White people (4–6). Moreover, minorities and people of low socioeconomic status have been found to underutilize eye care (30, 31) and barriers to healthcare often disproportionately affect these same populations (31–33).

About 12.4% of adults in Chicago have diabetes (34), mirroring the national prevalence of 11.3% (35). The University of Illinois Hospital & Health System (UI Health) is a safety net serving residents of communities who primarily identify as racial and ethnic minorities. In these communities, the prevalence of diabetes has been found to be over 30% higher compared to citywide estimates (36). As diabetes rates continue to increase in UI Health’s primary service area (PSA), diabetes-related complications require greater attention in healthcare delivery models. We developed a partnership between the Department of Family & Community Medicine (FM) and the Department of Ophthalmology and Visual Sciences (OVS) at UI Health to establish a care delivery model to integrate an AI-based screening program using the autonomous EyeArt EyeScreen AI system (Eyenuk, Inc) into a primary care setting. This screening was selected to provide immediate results in office so that patients can be educated and scheduled for an eye exam before leaving their primary care visit. In addition to the AI reading, an eye care provider can also grade images for other pathology. The overall goal of this program is to increase the number of patients with diabetes screened for DR, and to identify patients at high risk of vision loss and streamline their referral process into the ophthalmology clinic.

### Human-centered design

While interest in developing AI algorithms in healthcare has gained significant traction in recent years, most research has focused on examining its technical capabilities, revealing a gap in understanding how human factors affect implementation – an accurately predictive AI algorithm does not always translate into success in a real-world clinical environment (37, 38). Human-Centered Design (HCD), or “design thinking,” may help to understand real-world context and behaviors of individuals, engage stakeholders, and rapidly prototype and test solutions (39). HCD offers a methodology that allows researchers to explore why variation exists in order to create solutions that are integrated within the needs of various stakeholders (40). The iterative nature of HCD enhances usability and avoids unintended consequences by involving intended users throughout the entirety of the design process, gathering feedback to prioritize user needs using qualitative assessments and interviews (41). Beede and Baylor et al., the first human-centered observational study of a deep learning system deployed in a clinical setting, models the benefit of using HCD to evaluate AI systems, as this methodology helps understand end user needs and their environment, demonstrating how contextualizing these factors is key to the successful implementation of AI technology and acknowledging how success in a clinical environment cannot just depend on the accuracy of the technology alone (42). While traditional research methods aim to control for variation to demonstrate if an intervention changes behavior or improves other outcomes, in this paper, we describe using an adapted UK Design Council’s Double Diamond model to understand different stakeholder needs in the context of implementing an AI based DR screening program in a primary care clinic at UI Health.

## Context

### Setting

UI Health serves residents of communities who primarily identify as racial and ethnic minorities. These communities have greater rates of unemployment, uninsured individuals, and poverty than the average in the State of Illinois and U.S. (43). A fundus camera was installed within the University Village FM clinic. The FM clinic at UI Health provides primary care services, including offering preventive health screenings and managing long-term medical conditions such as diabetes.

### Participants

**Figure 1:**
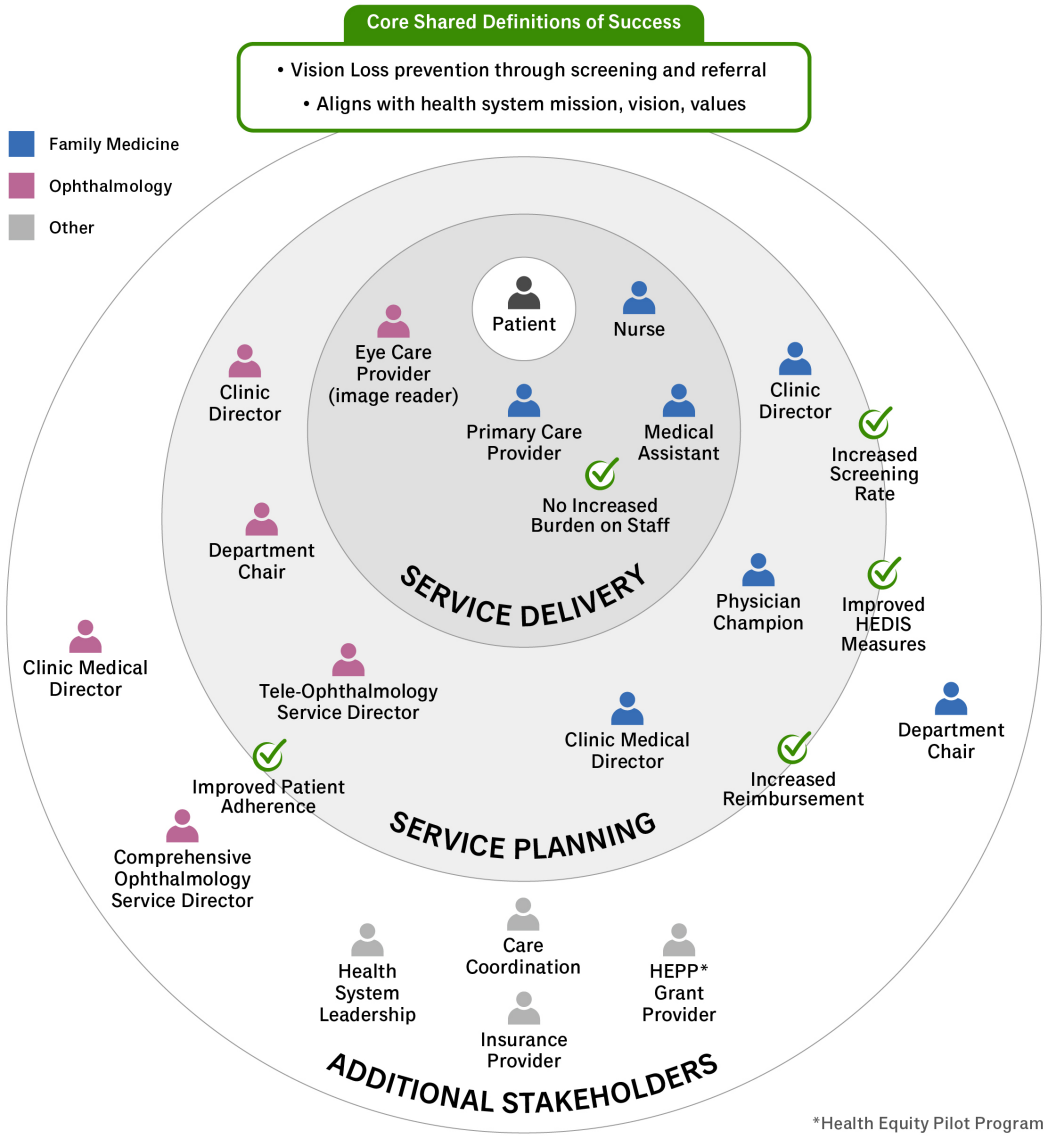
Key stakeholder map and definitions of success.

A key step in applying HCD in healthcare settings is to identify the individuals impacted by the problem or intervention in question. The target of this intervention includes any patient with diabetes presenting for a primary care visit who has not had a dilated fundus examination in the prior 12 months. Using estimates from the electronic health record, of the 1,476 patients with diabetes who completed an FM visit between 6 February 2022 and 6 February 2023, 822 (56%) were overdue for DR screening. While intervention planning was initiated by OVS and FM leadership, key stakeholders responsible for adopting and sustaining this screening include medical assistants, nurses, primary care providers, and image readers. Additional stakeholders are included in Figure 1.

## Programmatic elements

Human-centered design uses participatory mixed methods, rapid prototyping and iterative field testing to guide the delivery of novel health products or delivery strategies (44–47). HCD aims to involve key stakeholders early in a process to ensure that results meet the needs of the people it is meant to serve (48).

### Double diamond model

**Figure 2:**
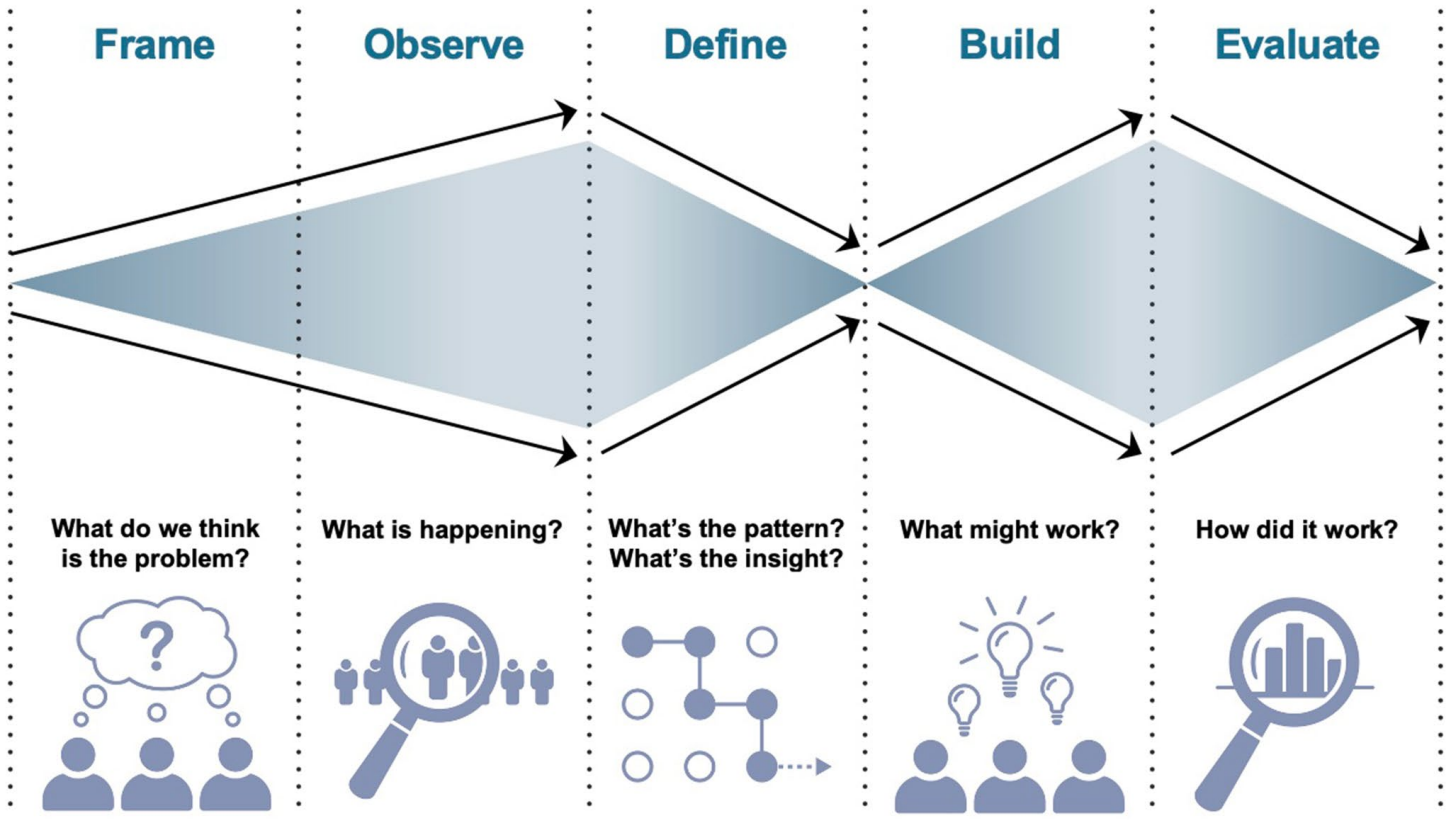
The double-diamond design model in healthcare delivery.

The Double Diamond Model is a framework that helps to guide the design process (49). While used in the design world since 2004, this framework has recently been used in healthcare research (50–52) and was selected in this work because it provides a simplified method for exploring a problem (i.e., divergent thinking) and taking a focused action (i.e., convergence) to come up with a solution (53). It is easily applicable and users are recommended to modify this model to suit individual project needs (53, 54). While the original model includes 4 phases (i.e., discover, define, develop, deliver), in healthcare delivery, this includes 5 phases: frame, observe, define, build, and evaluate, an adaptation of the original four phase process. In the frame and observe steps, context is explored as broadly as possible to gain understanding of the problem. In the define phase, the problem is reframed based on additional knowledge gained from the first two steps, and researchers converge upon a set of defined design principles to inform solution development. In the second diamond, a wide array of possible solutions are considered and prioritized to meet the needs of the problem. In each phase, methods of HCD are employed (e.g., key stakeholder interviews, contextual inquiry, observation, protocol development, multiple cycles of iteration) to come up with a viable, feasible, desirable program, key measures of HCD (55). Finally, these solutions are evaluated in live context, constituting a second convergence phase. Figure 2 illustrates the double diamond model.

#### Frame

The first step in designing the process for healthcare delivery is to frame the problem or goal of research. Initially, the project began with the question of: How do we *successfully* implement AI DR screening within the FM clinic? A key element of HCD is conducting stakeholder interviews to frame what broad terms like success entail in practice. From the initial stakeholder interviews, the following insights were extracted:

- The primary indicator of success harmonized among stakeholders at all levels was the shared goal of vision loss prevention through screening and referral.
- It is important to note that each stakeholder may have a different reason for wanting to implement this screening program, where success was defined at the department level. In FM, a successful screening program would also help them meet clinical target metrics for their patients, provide more holistic services to patients with diabetes, and generate revenue through reimbursement and improved Healthcare Effectiveness Data and Information Set (HEDIS) scores, a performance-based incentive to improve care quality (56). In OVS, a successful screening program would increase adherence to obtaining eye exams in patients with diabetes. The metric of success for the program would include the number of patients completing comprehensive eye exams.
- To prevent these differences from resulting in any kind of misalignment between the departments, multiple alignment meetings facilitated by the Institute of Healthcare Delivery Design were undertaken to ensure that both departments agreed on the ultimate mission of the program to maximize patient adherence to comprehensive eye exams and to use the screening as a triage tool to prioritize the order in which patients will be seen. Workflow decisions and education points were put in place to reflect that mission.

There are two approaches to implementation: forward mapping and backward mapping (57). Forward mapping begins at the top, with decisions being made at higher levels, and details being filled in later in the process. Although forward mapping might allow for more rapid development and implementation, a weakness of this process lies in the assumption that leadership controls the process that affects implementation. This is why many interventions fail; assumptions made by leadership in the early stages of development may not hold true in the lived experience of the workflow. While this program was created from the recommendation of leadership, our aim was to focus on the key stakeholders responsible for day-to-day activities in order to assure buy-in, adoption, and sustainability of this program. Backward mapping recognizes that leadership has a stake in the program, but rather than starting at the top, it focuses on a specific behavior at the frontlines of the implementation process that generates the need.

As described by Elmore, backward mapping offers a standard of success that is conditional (57). It is important to note that success is defined differently by each stakeholder and may even vary by context of use. For this reason, we identified key stakeholders to measures of success to understand the context within our health system (Figure 1). Once key stakeholders are identified, it is critical to create an implementation strategy with input from all stakeholders paying particular attention to this core team.

The second key finding from considering definitions of success was that success may look different to the different stakeholder groups within FM. For example, while all groups would value providing excellent patient care and prevent vision loss, the financial benefits and clinical target metrics of the program would likely be valued by clinic leadership but not by the staff members who would largely execute the program. Staff members would likely consider a successful program to be one that did not introduce workflows that were a high burden to carry out, including those for the screening, patient education, documentation, or scheduling. Because the success of the program hinged so much on the work of clinic staff, a successful implementation had to make their need for a low-burden program core to the program design.

#### Observe

Following the initial problem framing, design methodology seeks to further understand stakeholder needs and other critical components of the problem space via observation. Design emphasizes qualitative methodologies, particularly ethnography, to understand the context in which the problem space, and eventually the intervention, will be situated (58, 59). Contextual inquiry in the form of observations and semi-structured interviews allow design researchers to build a description of the setting, which includes not only observed behaviors, but also the context in which said behaviors occur (60). When contextualized, solutions can be calibrated to align with expected behaviors to increase the likelihood of uptake. One framework stratifies components into Activities, Environments, Interactions, Objects, and Users (AEIOU) (61).

The first human-centered observational study of a deep learning system deployed in a clinical setting evaluated the implementation of AI based DR screening in Thailand clinics by conducting observations and interviews to understand patient and nurse needs in order to improve the screening experience (42). For example, through pre-deployment observations, they uncovered that nurses already felt burdened by the existing patient volume, expressing concern that adding AI screening would add to their workload (42). By conducting post-deployment observations, they were able to identify that ungradable AI images were a significant contributor to increased work burden and nurse frustration as ungradable photos required multiple time consuming retakes until the image was satisfactory for the algorithm, ultimately resulting in adjustment of the study protocol to involve ophthalmologists in evaluation ungradable images later, instead of relying on nurses to retake the image at the same visit (42).

Just as Beede and Baylor et al. conducted field observations and interviews to elucidate existing clinic workflows and explore nurse and patient expectations prior to the deployment of AI screening (42), we conducted observations within the Department of Family Medicine that revealed the centrality of medical assistants (MAs) to the implementation of new workflows. As patients’ main touchpoint throughout the clinical encounter, MAs guide patients from the waiting room and situate them. Any application of the DR screening would fall within the MAs’ purview, and therefore screening implementation would have to properly educate MAs as to the value of the program, create buy-in, as well as co-exist with competing responsibilities of rooming multiple patients, attending to message pools, and stocking rooms. The aim of these pre-deployment observations is that they will inform the deployment and subsequent iterations of the AI screening program to maximize patient and provider experiences.

#### Define

Following the initial two steps of the design process, key insights are extracted from observations in the define phase. Reflection following the design activities outlined above yielded a number of design requirements or “design principles” (i.e., solution functions and features) necessary for the successful implementation and sustainability of the screening program (62, 63). Although individual design principles, when stated, may appear self-evident, the set of identified design principles in concert frame prioritized, non-negotiable components of an initiative. To this end, six design principles were identified: (1) Front-line clinician and staff buy-in; (2) Efficient and straightforward workflows; (3) Tangible and visible operational protocols; (4) Educational materials; (5) Consistent delivery of key messages; (6) Facilitating the scheduling of eye exams.

First, front-line clinician and staff buy-in would be vital for successful implementation. Top-down implementation of a workflow without adequate buy-in may result in initial success but substandard sustainability. Moreover, front-line buy-in would allow for comprehensive understanding of key messages and proper prioritization of the novel initiative in an already busy schedule.

Second, designed workflows would have to be efficient and straightforward so as to build on the first design principle. This streamlined nature would accordingly value the time of clinicians and staff, allowing clinicians to practice at the top of the license and be available when necessary. For example, workflows may emphasize the importance of clinicians delivering positive results of eye disease but not negative results. In addition, clinicians may not be required to spend several extra minutes for each patient educating patients on the screening when staff would be able to fulfill this duty. This second principle in turn leads to the third design principle: tangible and visible operational protocols for staff. Scripts, scheduling protocols and guides, and screening ordering protocols would be made available for all staff both for initial training and for continued education and maintenance of skills.

On the patient end, the fourth and fifth identified design principles refer to educational materials for patients and delivered key messages, respectively. Based on interviewing clinic staff experienced in interfacing with patients, it was suggested that having educational handouts would be important to both reinforce information in the clinic and to also provide patients with material to review at home in case they do not choose to undergo screening at the visit. Consistent delivery of these key messages would underpin the communication strategy for patients requiring comprehensive eye exams for DR.

The patient experience culminates in the sixth design principle—patients with an abnormal or ungradable result would be granted the opportunity to schedule eye exam appointments during the check-out process of the initial clinical encounter. Based off of electronic health record estimates, of the 1,467 patients with diabetes seen in Family Medicine Clinic between 6 February 2022 through 6 February 2023, 822 (56%) were overdue for their DR Screening Health Maintenance Topic. Family medicine referred 323 patients to ophthalmology for a diabetes-related exam, of which an estimated 160 (50%) are predicted to schedule a visit and 130 (40%) are predicted to ultimately complete the visit. The current state also places the onus on patients to schedule their eye exam after they have exited the clinical encounter, which may influence adherence rates. While data associating AI screening with adherence is limited given how many factors may influence a patient’s ability to receive follow-up care, in a prospective study at a primary care clinic, adherence to follow-up ophthalmic evaluation was 55.4% at 1-year following implementation of an AI screening program for DR, compared to the clinic’s historical adherence rate without AI screening of 18.7% (*p* < 0.0001, Fisher’s Exact Test) (25). While these rates were obtained in separate studies, it is unclear if AI screening alone can explain the increase in adherence. In the AI screened cohort, all patients with referable DR were immediately referred to ophthalmology during the primary care visit through the electronic health record and pro-actively received multiple phone calls reminding them to schedule an appointment, whereas the historical cohort was analyzed retrospectively and patients were either encouraged to follow-up with an ophthalmologist or referred without mention of a pro-active calling protocol to remind patients to schedule an appointment (64). Additionally, the authors suggest that the immediate result provided by AI screening may have had significant effects on increasing rates of follow-up (25). Tying the scheduling process with the check-out process may not only remove energy barriers to setting up an appointment, but may also allow patients to manage their health at a time when they are most mentally primed to think proactively (i.e., immediately following screening results).

#### Build

Using the above design requirements, we began the process of brainstorming potential interventions (65). Brainstorming sessions result in ideas that are narrowed based on their applicability to design opportunities and feasibility. Narrowed ideas are then developed into early concepts or prototypes which undergo iterative cycles of testing with users in the Implementation phase. A concept or prototype offers a tangible representation with which to engage stakeholders in imagining future states that do not yet exist, making it a valuable tool in healthcare research (66–68). This is a mechanism for collecting real world design requirements that only come out through working collaborations. They accelerate desirable, feasible, viable solutions by integrating the people who will ultimately be using them. Many subtle but significant details, specifically usability, are resolved through this process. Paper and experience prototypes have both been shown to accelerate the development process and provide a highly optimized output that is risk-reduced with higher rates of adoption (67, 68).

**Figure 3:**
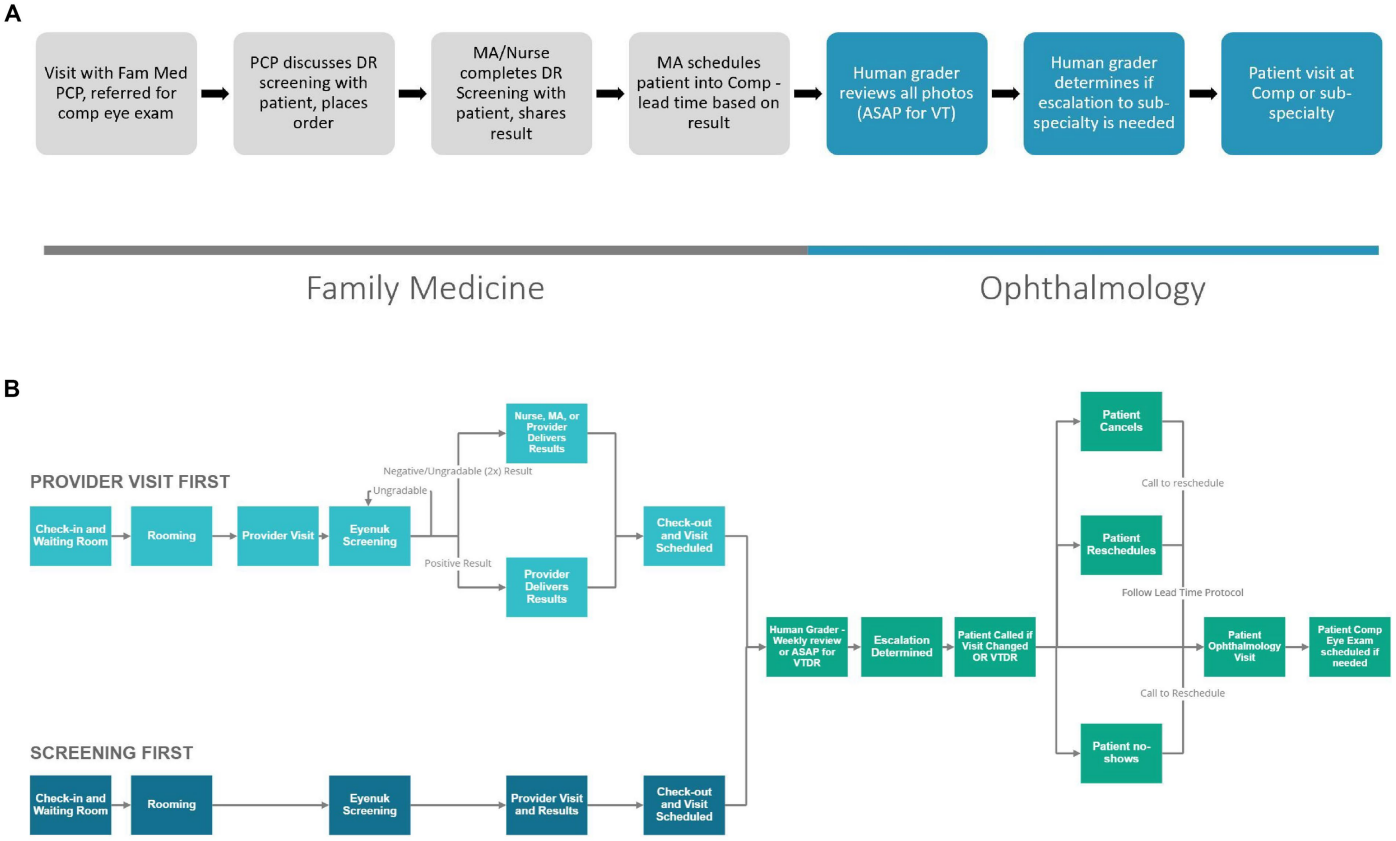
**(A)** Early workflow concept. **(B)** Updated workflow based on stakeholder feedback.

When creating the clinic workflow for this program, we began with a proposed workflow to share with all stakeholders. The greatest workflow change compared to current referrals to ophthalmology was that patients would be leaving the appointment in FM with an ophthalmology appointment in hand. The responsibility of scheduling was moved from the patient to the medical assistant, so we looked to the medical assistant and clinic director to assure this work was possible and would not delay the clinic workflows in FM. The original proposed workflow (Figure 3A) shows a general structure of the workflow used to generate feedback from stakeholders. With input from stakeholders within the FM and ophthalmology leadership and FM core team, the workflow continued to be reiterated. Figure 3B shares an updated concept. This workflow shows additional details based on when the photo is taken, who can provide results to the patient (based on changes made in the design phase) and shows prioritization of patients with positive results to ensure timely follow-up within the ophthalmology clinic.

**Figure 4:**
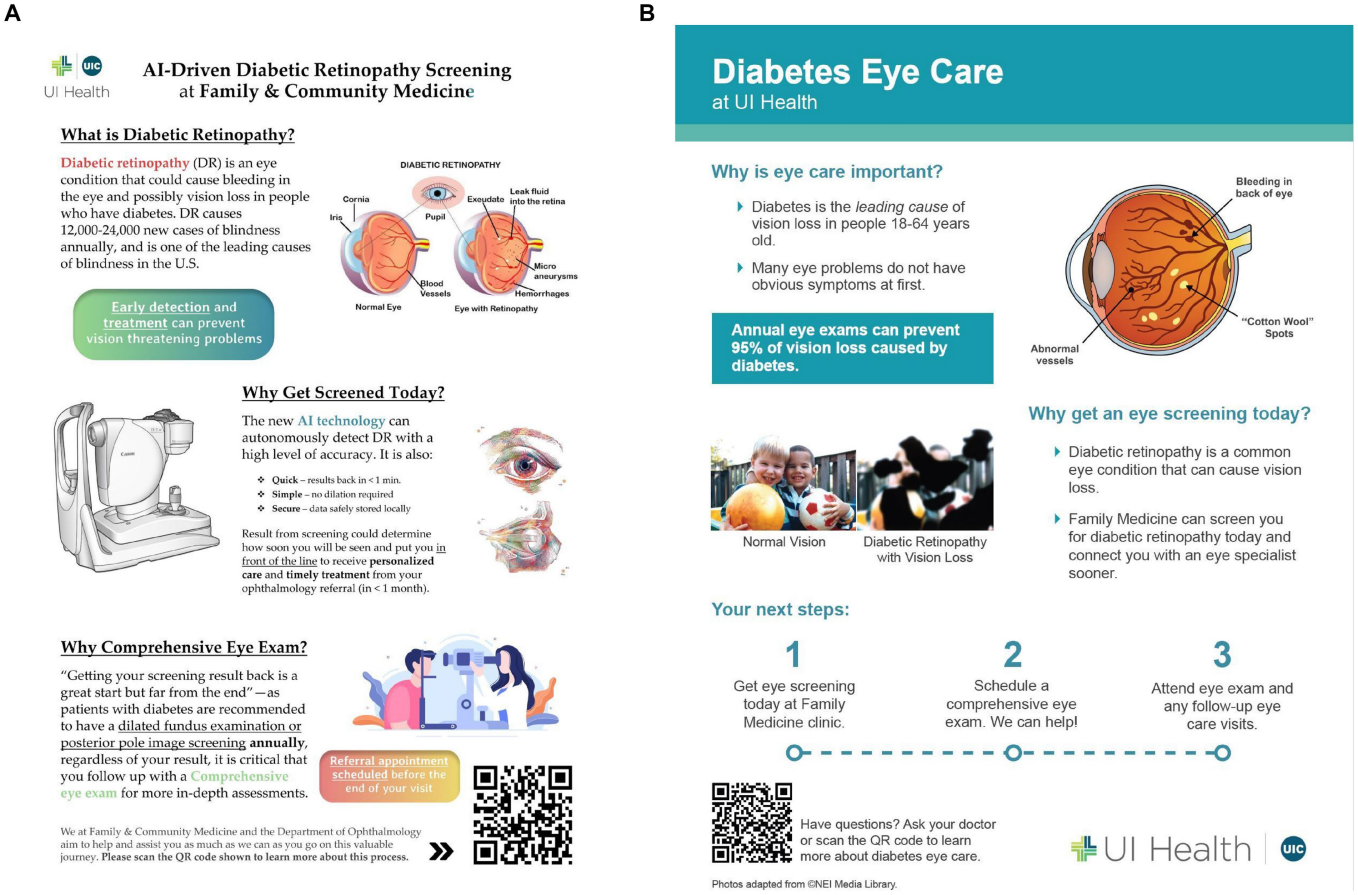
**(A)** Early patient educational brochure. **(B)** Updated patient educational brochure.

Another important concept discussed within the define section was the creation of education tools for patients, staff, and providers in order to promote consistent messaging among all stakeholders. These messages would include that DR is a common complication of diabetes that can threaten vision, that a comprehensive eye exam is recommended to evaluate for other pathology, and that screening would help the clinic expedite the patient visit to the Department of Ophthalmology. An early concept of an educational brochure for patients is shown in Figure 4A. With feedback from stakeholders, it was suggested to simplify this document to make it easy to read with key points shared in one familiar place. These educational and motivational materials would be augmented with key messages delivered to patients throughout the clinical encounter. Figure 4B shows an updated educational tool that describes DR, why eye screening is critical in patients with diabetes and next steps to make eye health a priority.

#### Evaluate

In the last step, the concepts are tested and evaluated. This step continues through several iterations with continuous feedback from end-users. Measurements can include key metrics for implementation research: acceptability, adoption, appropriateness, feasibility, and adaptation (69). Other measures specific to this intervention include post-implementation referrals, appointments scheduled, and appointments completed. Additional data may be derived from surveys of patients, staff, and providers, in addition to post-implementation interviews and observation. Implementation of the system followed educational sessions during FM staff and provider meetings. While the concept for implementation has been evaluated through multiple cycles of iteration and feedback sessions, data evaluating post-implementation referrals and appointment adherence are pending future study.

### Implications

In this paper, we focus on the efforts made prior to the deployment of an evidence-based intervention into routine clinical care, which falls within the implementation science (i.e., T3) arm of the translational science spectrum (70). What we learned from the double-diamond approach is that implementation of new technology in a healthcare system is not one size fits all. What works for one population or setting may not work for the next. Moreover, without key stakeholder input, an intervention is less likely to serve its intended audience. It is critical to spend the time identifying stakeholders, key design requirements, and sharing concepts or prototypes to strengthen a program at its inception. Future work will include a post-implementation analysis.

## Data availability statement

The original contributions presented in the study are included in the article/supplementary material, further inquiries can be directed to the corresponding author.

## Author contributions

AS, CB, HM, AL, and RVPC contributed to the conception and design of this work. AP, DD also contributed to the design of this work. AS wrote the first draft of the manuscript. AS, CB, AP, DD, PB wrote sections of the manuscript or developed the figures. All authors contributed to the article and approved the submission.

## Funding

This work was supported by funding from NIH/NEI K12 EY021475 (Scanzera), NIH/NEI P30 EY001792, NIH/NEI R01 EY029673 (Chan), Health Equity Pilot Project, the Cless Family Foundation, and an unrestricted grant to the Department of Ophthalmology and Visual Sciences from Research to Prevent Blindness. The funders were not involved in the design, collection, analysis, interpretation of data, writing of this article, or the decision to submit the publication.

## Conflict of interest

R.V. Paul Chan discloses the following 1) Alcon (Consultant); 2) Genentech (Consultant); 3) Siloam Vision (Owner/Equity).

The remaining authors declare that the research was conducted in the absence of any commercial or financial relationships that could be construed as a potential conflict of interest.

## Publisher’s note

All claims expressed in this article are solely those of the authors and do not necessarily represent those of their affiliated organizations, or those of the publisher, the editors and the reviewers. Any product that may be evaluated in this article, or claim that may be made by its manufacturer, is not guaranteed or endorsed by the publisher.
